# Media from macrophages co-incubated with *Enterococcus faecalis* induces epithelial cell monolayer reassembly and altered cell morphology

**DOI:** 10.1371/journal.pone.0182825

**Published:** 2017-08-09

**Authors:** Natalia Belogortseva, Monika Krezalek, Kristina Guyton, Christine Labno, Valeriy Poroyko, Olga Zaborina, John C. Alverdy

**Affiliations:** 1 Department of Surgery, University of Chicago, Chicago, Illinois, United States of America; 2 Integrated Light Microscopy Core Facility, University of Chicago, Chicago, Illinois, United States of America; 3 Department of Medical Oncology and Therapeutics, Research City of Hope Comprehensive Cancer Center, Duarte, California, United States of America; Indiana University School of Medicine, UNITED STATES

## Abstract

Signal exchange between intestinal epithelial cells, microbes and local immune cells is an important mechanism of intestinal homeostasis. Given that intestinal macrophages are in close proximity to both the intestinal epithelium and the microbiota, their pathologic interactions may result in epithelial damage. The present study demonstrates that co-incubation of murine macrophages with *E*. *faecalis* strains producing gelatinase (GelE) and serine protease (SprE) leads to resultant condition media (CM) capable of inducing reassembly of primary colonic epithelial cell monolayers. Following the conditioned media (CM) exposure, some epithelial cells are shed whereas adherent cells are observed to undergo dissolution of cell-cell junctions and morphologic transformation with actin cytoskeleton reorganization resulting in flattened and elongated shapes. These cells exhibit marked filamentous filopodia and lamellipodia formation. Cellular reorganization is not observed when epithelial monolayers are exposed to: CM from macrophages co-incubated with *E*. *faecalis* GelE/SprE-deficient mutants, CM from macrophages alone, or *E*. *faecalis* (GelE/SprE) alone. Flow cytometry analysis reveals increased expression of CD24 and CD44 in cells treated with macrophage/*E*. *faecalis* CM. This finding in combination with the appearance colony formation in matrigel demonstrate that the cells treated with macrophage/*E*. *faecalis* CM contain a higher proportion progenitor cells compared to untreated control. Taken together, these findings provide evidence for a triangulated molecular dialogue between *E*. *faecalis*, macrophages and colonic epithelial cells, which may have important implications for conditions in the gut that involve inflammation, injury or tumorigenesis.

## Introduction

Dynamic crosstalk between intestinal epithelial cells (IECs), the microbes that colonize their apical surface and the surrounding local immune cells is necessary to maintain intestinal homeostasis [1]. An imbalance in this triangulated interaction may disturb intestinal epithelial integrity resulting in multiple downstream consequences including inflammation, oncogenesis and the development of primary tumors into metastasis [2–4]. Evidence suggests that bacterial derived proteases may play a primary role in this process [5]. For example, *Enterococcus faecalis* metalloprotease GelE can directly compromise the intestinal epithelial barrier [6] and induce inflammation through surface-associated lipoproteins [7]. Previous work from our laboratory has demonstrated that *E*. *faecalis* can activate macrophage matrix metalloprotease MMP-9 in a GelE/SprE dependent manner leading to disruption of anastomotic healing [8]. Given the close proximity of the intestinal epithelium and macrophages, here we sought to explore whether a co-interaction between *E*. *faecalis*, a common member of the intestinal microbiota, and macrophages can alter epithelial monolayer integrity/cell morphology. We also sought to define the contribution of secreted proteolytic enzymes of *E*. *faecalis*, i.e GelE and SprE, in this process.

## Materials and methods

### Bacterial strains

In this study were used wild type *E*. *faecalis* V583 and its derivative mutants Δ*gelE*Δ*sprE* and complemented mutants Δ*gelE*Δ*sprE/gelE*+*sprE* provided by Lynn Hancock [8, 9]. All strains were stored in 10% glycerol stock at −80°C. Only cells freshly plated from stock were used in experiments. Cells from stock were plated onto tryptic soy broth (TSB) plates, grown overnight at 37°C.

### Co-incubation of murine macrophage with E. faecalis strains

The murine macrophage cell line J774 (J774A.1, ATCC) was cultured in DMEM (Invitrogen) supplemented with 10% fetal bovine serum. *E*. *faecalis* strains were grown in THB for 6h to OD_600_ of approximately 1.5–2. Bacterial density was then adjusted by serial dilution in THB to OD_600_ = 1, which corresponds to approximately 5 × 10^8^ cells/ml as measured by plating 10-fold serial dilutions. Before co-incubating *E*. *faecalis* strains with macrophages, 10 ml of the macrophage suspension of 1x10^6^ cells/ml (i.e a total macrophages cell dose of 1x10^7^) was seeded onto a 10mm cell culture dish. In all experiments, we used macrophages infected with *E*. *faecalis* at low multiplicities of infection (MOI), namely, a bacterial cell suspension (100μl) with an OD_600_ = 1.0 (5 × 10^8^ cells/ml) was added to 10 ml serum free DMEM (total bacterial cells = 5x10^7^) and after macrophages attachment, medium was replaced on DMEM containing *E*.*faecalis* strains. This corresponded to a MOI = 5 (5x10^7^**:** 1x10^7^). The bacterial cell density during 1, 2, 3, 4, 5 h was measured by serial dilutions of plated bacteria. The concentration of bacteria (CFUs) was increased approximately 3 fold following 5hrs of coincubation (S1 Fig). After 5h of co-incubation, the conditioned medium (CM) was collected, sterilized by their filtration trough 0.22 μm pore membrane, and then applied onto epithelial monolayers. For controls, CM from macrophages alone and media from *E*. *faecalis* alone were used.

### Cell viability assay

In order to determine if macrophage viability was compromised by co-incubation with *E faecalis*, we measured lactate dehydrogenase (LDH) release following 5 hrs of coincubation of *E*. *faecalis* with J774 macrophages. LDH was measured using LDH assay kit (CytoTox 96 Non-Radio, Promega) according to the manufacturer’s instructions. The percentage of macrophage cytotoxicity was calculated as follows: (sample absorbance-background absorbance)/(maximal absorbance-background absorbance) x 100. Results indicated that LDH activity (% cytotoxity) was negligible in culture supernatant of J774 macrophages co-cultured with *E*. *faecalis* WT at MOI = 5 for 5h (S2 Fig).

### Epithelial cell culture and treatment

**Fig 1:**
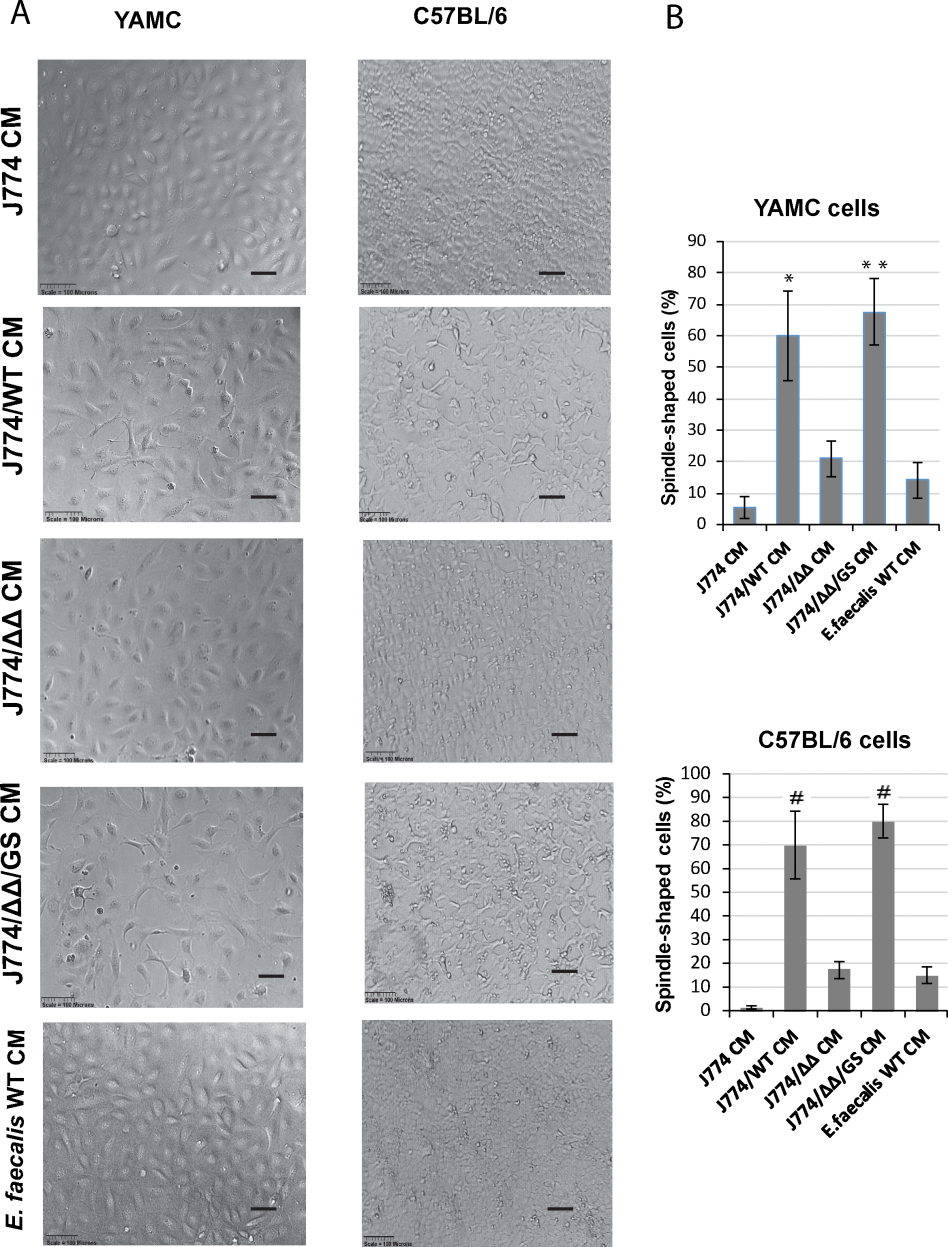
Uniformity of morphological changes in both YAMC and C57BL/6 cells in response to conditioned media isolated after exposure of J774 macrophages to *E*. *faecalis* (Scale bars– 100 μm). **(A)** Phase contrast microscopy images of primary mouse colon epithelial cells incubated with J774/*E*. *faecalis* CMs demonstrates reassembly of epithelial monolayer and morphological changes of adherent epithelial cells when J774/*E*. *faecalis* (GelE/SprE) CMs were used for co-incubation with J774; **(B)** The percentage of flattened spindle-shaped adherent epithelial cells. Four fields of phase contrast images from 3 independent experiments were taken for total cell count. Determination of morphological changes was based on cobblestone or spindle cell shape. The number of spindle-shaped epithelial cells in J774/WT CM and J774/ΔΔ/GS CM groups was significantly higher compared to J774/ΔΔ CM. Mean values are graphed and error bars represent standard error of the mean (SEM). Statistical analysis was performed using the one-way ANOVA with Bonferrony adjusted *p*-value for multiple comparisons where *P = 0.002, **P = 0.001 and ^#^*P* = 0.001 as compared to J774/ΔΔ CM, n = 3.

Young Adult Mouse Colon (YAMC) cells, an immortalized cell line, were kindly provided by Dr. Musch (University of Chicago) and cultured as described [10]. C57BL/6 mouse primary colonic epithelial cells were obtained from Cell Biologics (US, Chicago, Cat.No C57-6047) and grown according to company recommendations. YAMC cells were grown at 33°C and C57BL/6 cells at 37°C in a humidified 5% CO2 atmosphere. We used the C57BL/6 cells at passage 3–8. For all cell treatments, cell growth media was replaced with J774/*E*. *faecalis* CMs or with appropriate control CMs or with the above indicated CMs in the presence of a MMP9 inhibitor I (10μM) (Calbiochem, Cat. # 444278). After 18 h of co-incubation the cells and their CMs were analyzed. The percentage of spindle-shaped cells was quantified as described in legend to Fig 1. Total and live cell counts (with trypan blue) were measured using Automated Cell Counter (Bio-Red TC20).

### Fluorescence microscopy

***a)*** For fluorescence staining, cells were fixed in 4% paraformaldehyde (PFA) for 20 min at room temperature followed by PBS washes 3 times. Cells were stained overnight at 4°C for epithelial markers with mouse anti-E-cadherin (1:100; Novus Biological) and rabbit anti-ZO1 (1∶250; Invitrogen) and for mesenchymal marker vimentin with rabbit anti-vimentin (1:100; Cell Signaling). Cells were washed three times with PBS and incubated for 1 h at room temperature (RT) treated with goat anti-mouse IgG Alexa-Fluor-488 (Abcam) and donkey anti-rabbit IgG Alexa-Fluor-488 (Invitrogen). Fluorescence of the images was quantified using the ImageJ software and expressed as the area of ZO-1 and E-Cadherin per cell. Rhodamine phalloidin (Cytosceleton) was used to stain the actin cytoskeleton. To detect apoptotic cells, cells were stained with FITC-annexin for phosphatidylserine and fixed with PFA. ***b)*** Whole mount fluorescent staining of colonies formed in matrigel was performed according to previously described protocols [11]. Prolong Gold antifade reagent with DAPI (Invitrogen) was used for confocal imaging. Images were obtained using Leica fluorescence microscope, SP8 laser scanning confocal (Leica microsystem, Mannheim, Germany). 3D reconstructions of deconvoluted image stacks were carried out using Imaris x64 Version 7.3.1 software (Bitplane).

### Western blot

Cells were lysed with ice-cold lysis buffer (50mM Tris-HCl, pH 7.4, 150mM NaCl. 0.5% Triton X-100 and PMSF.) After 15 min of incubation on ice, the lysates were centrifuged at 10000 rpm for 15 min to remove debris. The supernatants (15 μl that contained ~30 μg of protein) were boiled for 5 min with Laemmli sample buffer, electrophoresed through 10% SDS-polyacrylamide gels and then transferred onto PVDF membranes (Immobilon-P, Millipore). The membranes were blocked in Tris-buffered saline containing Tween 20 (pH 7.4) and 4% non-fat dry milk (Labscientific). Membranes were incubated with primary mouse anti E-cadherin (Novus Biological) followed by the corresponded HRP coupled secondary antibody (Cell Signalling) and developed using ECL Western Blotting Detection Reagents (G&E Helthcare). Densitometry analyses were performed using ImageJ software.

### Zymography assay

Zymography was performed as previously described [12] with some modification. Briefly, 7.5% SDS-PAGE gels containing 0.1% gelatin (Sigma) were prepared. The samples in SDS-sample buffer without reducing agent and without boiling were subjected to SDS-PAGE. After electrophoresis, gels were washed with 50 mM Tris-HCl buffer pH 7.4 containing 5 mM CaCl_2_, 1 μM ZnCl_2_ and 2.5% Triton X-100, for 1 hr at room temp on a rotating shaker to remove the SDS. The gels were then incubated overnight at 37°C with 50 mM TrisHCl buffer pH 7.4 containing 5 mM CaCl_2_ and 1 μM ZnCl_2_, and stained with 0.5% Coomassie Brilliant Blue G-250 dissolved in 30% ethanol plus 10% acetic acid for 30 minutes. Destaining was achieved by incubation in 30% ethanol plus 10% acetic acid. Following destaining, zones of enzyme activity were detected as regions of negative staining.

### Flow cytometry

Cells were harvested and 1X10^6^ fixed in 2% PFA for 15 min. at room temperature, then rinsed by FACS buffer (eBioscience) with the following antibodies or isotype control incubation for 1 h: PE/Cy7 anti-mouse CD44 (1.5:100) (BioLegend #103030), PE/Cy7 rat IgG2b (1.5:100) (BioLegend #400617), eFluor 450 anti-mouse CD24 (1.25:100) (eBioscience #48-0242-82), rat IgG2b eFluor 450 (1.25:100) (eBioscience #48–4031). Cells were washed and then re-suspended in FACS buffer for analysis. Flow Cytometry data acquisition was performed at the University of Chicago Cytometry and Antibody Technology Facility using a BD Biosciences LSR II. Data analysis was performed using FlowJo v.10.2 software.

### Matrigel colony assay

Cellular suspensions after treatment with 0.05% trypsin for 1–2 min were filtered through 30-μm filter (Cetrics). Single cell suspensions (50 μl containing ~ 1000 cells) were mixed with 50μl of matrigel (Matrigel Matrix, Growth Factor Reduced, #356231, Corning) and 50 μl of the cell suspension in matrigel was plated into 1 well of Nunc 8-well chambers-slide and grown in medium supplemented with 10% FBS. After 1 week of incubation the colonies were identified.

### Treatment of mouse colon tissue explants

All animal experiments were approved by the Institutional Animal Care and Use Committee at the University of Chicago (IACUC protocol 71744). Eight week old male C57B/L6 mice weighing 18-22g were used. Mice were sacrificed as per the guidelines of the University of Chicago Institutional Animal Care and Use Committee, using CO2 gas overdose followed by secondary cervical dislocation. The abdominal cavity was opened using aseptic technique and the entire colon isolated from the cecum to rectum. The colon was cut into eight 5mm x 5mm pieces which were then incubated for 6 hours with control J774 CM and J774/WT CM in replicates. Following incubation, tissues were fixed in 4% paraformaldehyde, stained with the epithelial marker rhodamine labelled WGA (Vector Laboratories) and mounted in Prolong Gold antifade reagent with DAPI.

### Statistical analysis

Mean values are graphed and error bars represent standard error of the mean (SEM). Statistical analysis was performed using the one-way ANOVA with Bonferrony adjusted *P* value for multiple comparisons. *P* value less than 0.05 was considered statistically significant.

## Results and discussion

### Conditioned media from J774 macrophages co-incubated with E. faecalis GelE/SprE producing strains induced morphological and cytoskeletal changes in primary mouse colonic epithelial cells

**Fig 2:**
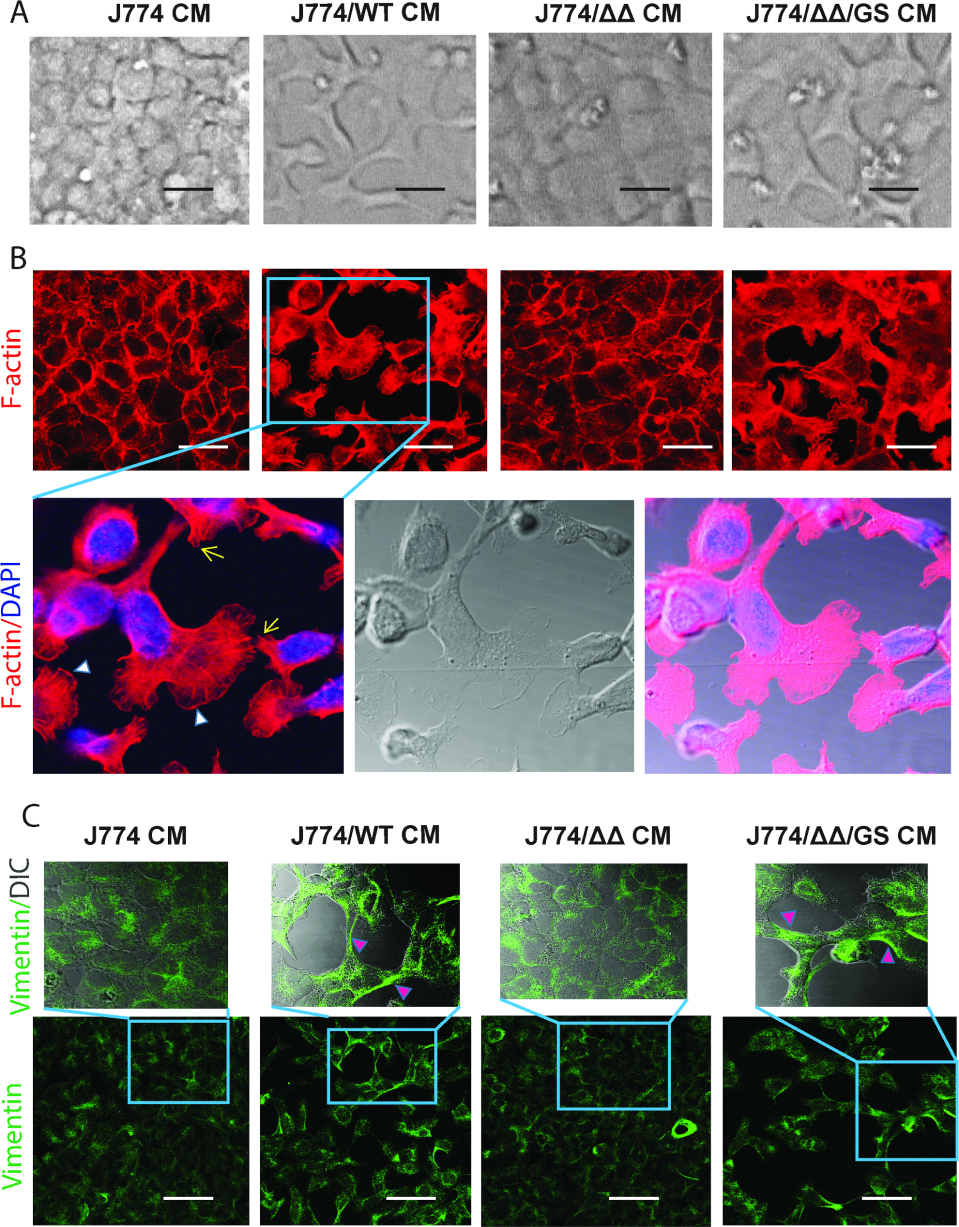
Morphological and cytoskeletal changes in mouse primary epithelial cells (C57BL/6) induced by conditioned media from J774 macrophages co-incubated for 18h with *E*. *faecalis* GelE/SprE producing strains. **(A)** Phase contrast microscopy images of primary mouse colon epithelial cells incubated with J774/*E*. *faecalis* CMs. Representative images of 10 experiments; **(B)** Immunostaining with rhodamine phalloidin for F-actin (red) and nucleus with DAPI (blue). Lamellipodia and filopodia formation are shown by white arrowheads and yellow arrows respectively; (**C)** Immunostaining for vimentin. Pink arrowheads indicate intermediate vimentin filaments. Scale bars– 20 μm.

In present study we used *E*. *faecalis* V583 wild type (WT), its mutant deficient in the GelE/SprE (*ΔΔ*), and the complemented mutant ΔGelEΔSprE/*gelE+spreE* (*Δ*Δ/GS) [8, 9]. Secretion and functionality of GelE and SprE by *E*. *faecalis* has been described in detail from our laboratory [8]. Mouse macrophages (J774) were incubated with *E*. *faecalis* strains for 5 hours. Conditioned medium (CM) was collected and depending on the *E*. *faecalis* strain, designed as J774/WT, J774/ΔΔ, J774/ΔΔ/GS CMs. The sterilized CMs were applied onto two types of intestinal cells: YAMC (young adult mouse colonic epithelium) or C57BL/6 mouse primary colonic epithelial cells (generated by Cell Biologics) grown on collagen-coated plastic. In all experiments, epithelial cells were cultured to form monolayers. After 18 hours of co-incubation of epithelial cells with CMs, phase contrast microscopy demonstrated that J774/ WT CM and J774/ΔΔ/GS CM induced clear reassembly of epithelial monolayers and striking morphological changes in adherent cells characterized by mesenchymal-like morphology in both epithelial lines (Fig 1). The images demonstrated uniformity in the morphological changes in both YAMC and C57BL/6 cells and these changes were not observed when epithelial cells were incubated with CM from J774 cells alone or CM from J774/ΔΔ or control media with *E*. *faecalis* alone. Transformed cells adopted a spindle-shaped morphology and some cells displayed a flat polygonal shape with multibranches (Fig 2A). Fluorescence staining of the actin cytoskeleton with rhodamine phalloidin demonstrated significant alterations in actin cytoskeleton organization (Fig 2B). Adherent cells with altered morphology exhibited marked filamentous filopodia, lamellipodia, and stress fiber formation at the ventral cell surface with the loss of cortical actin bundles. Other key cytoskeletal proteins such as vimentin also displayed reorganization. These cells demonstrated formation of vimentin intermediate filaments (Fig 2C). Vimentin expression has been shown to correlate with mesenchymal cell shape and motile behavior [13, 14].

These results demonstrate that the interaction of macrophages with GelE/SprE producing *E*. *faecalis* initiates dissolution of epithelial cell-cell contact with some cells undergoing cell extrusion whereas others remain as adherent cells forming F-actin basal protrusions that increase cell–matrix contacts- features consistent with cell migratory capacity [15–17].

### Exoproducts of the Macrophage/E. faecalis CMs affect intercellular junctions in epithelial monolayers

**Fig 3:**
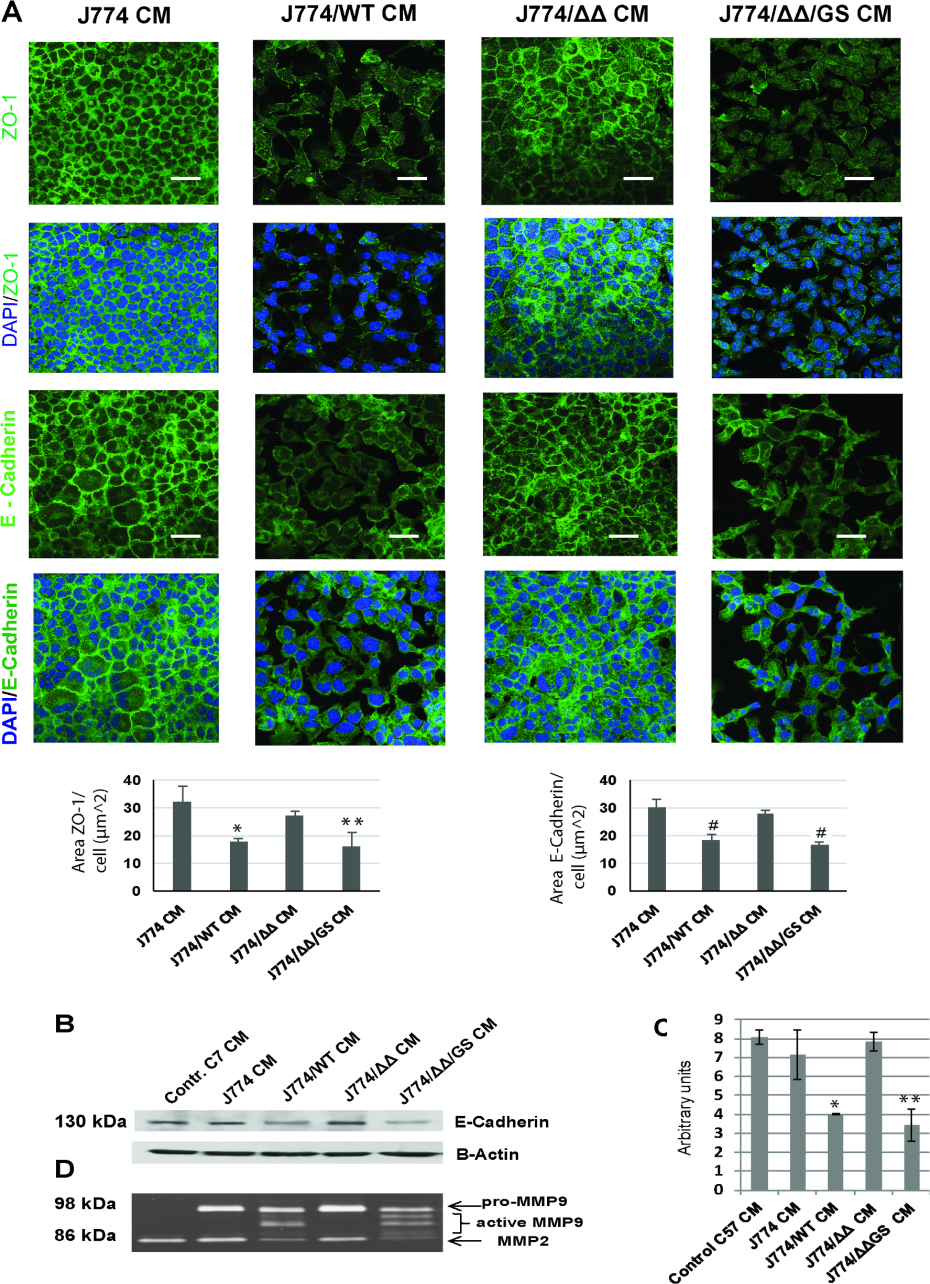
Changes in expression and localization of epithelial cell-cell junction proteins E-cadherin and ZO-1 after 18 h of treatment of epithelial cell monolayers with macrophage/*E*. *faecalis* CM. **(A)** Immunostaining for ZO-1 and E-cadherin. Scale bars– 20 μm; Area of ZO-1 and E-Cadherin fluorescence per cell in images in J774/WT CM and J774/ΔΔ/GS CM groups was significantly lower compared to J774 CM. Mean values are graphed and error bars represent standard error of the mean (SEM). Statistical analysis was performed using the one-way ANOVA with Bonferrony adjusted *p*-value for multiple comparisons where *P = 0.007, **P = 0.004 and ^#^*P*<0.001 as compared to J774 CM, n = 3. **(B)** Representative Western blot staining of E-cadherin in epithelial cells treated with CMs from macrophages co-incubated with *E*. *faecalis* strains. Results are typical of three independent experiments; **(C)** Expression of E-cadherin in epithelial cells treated with J774 CM and J774/*ΔΔ/GS* CM was significantly lower compared to J774 CM and consistent with fluorescent staining data for E-Cdherin. Statistical analysis was performed using the one-way ANOVA with Bonferrony adjusted *p*-value for multiple comparisons where *P = 0.005, **P = 0.002 as compared to J774 CM, n = 3. **(D)** Zymography of resulting epithelial cells CMs demonstrating the presence of active form of metalloproteinase MMP9 upon exposure to macrophage/E. *faecalis* CMs when GelE/SprE secreted strains (WT and ΔΔ/GS) were used.

**Fig 4:**
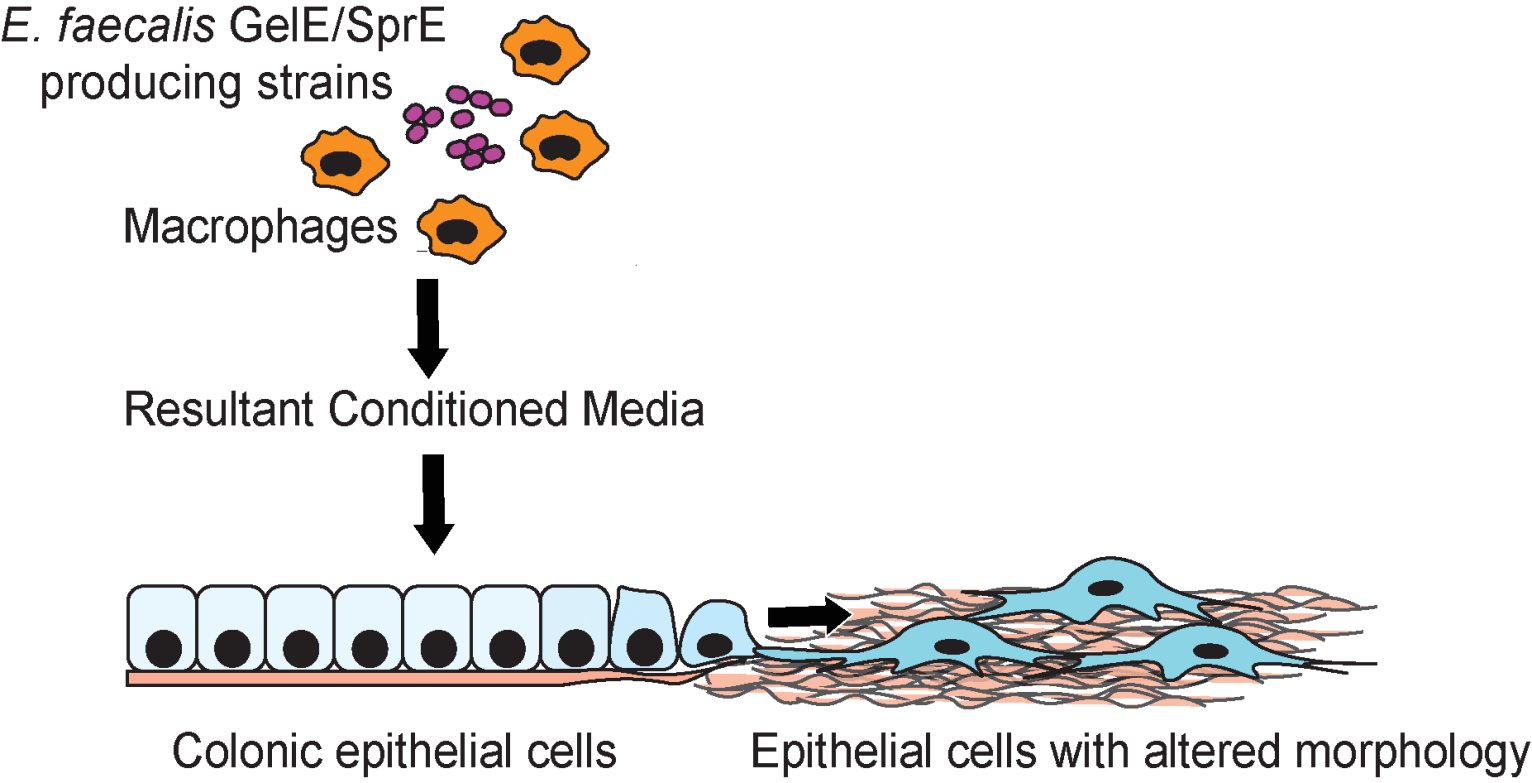
Proposed model of epithelial monolayer reassembly. Conditioned media from macrophages co-incubated with *E*. *faecalis* (GelE/SprE) leads to reorganization of epithelial cells, which adopt flattened and elongated shapes. These transformed cells exhibit marked filamentous filopodia and lamellipodia formation. Cellular reorganization is not observed when epithelial monolayers are exposed to CM from macrophages co-incubated with *E*. *faecalis* GelE/SprE-deficient mutants, to the CM of macrophages alone, or to *E*. *faecalis* (GelE/SprE) alone.

It is known that changes of epithelial cells morphology depend on cell-cell and cell-substrate adhesion [18]. Cell-cell contact is maintained via intercellular junction proteins which through connections to the actin cytoskeleton, coordinate cellular organization, maintain the integrity of epithelial monolayer and control phenotypic transition of epithelial cells [19, 20]. Treatments of epithelial cells monolayers with macrophage/*E*. *faecalis* conditioned media led to disruption of monolayer integrity when *E*. *faecalis* GelE/SprE producing strains were used. Immunofluorescence of the tight junction protein ZO-1 and adherent junction protein E-cadherin demonstrated a reduction in their expression and a change in their distribution among transformed cells (Fig 3A). Western blot (Fig 3B), and its densitometry analysis (Fig 3C and S3 Fig) also showed slightly decreased expression of E-cadherin in transformed cells. Previously it has been demonstrated that MMP9 catalyzes E-cadherin ectodomain shedding [21, 22] and contributes to epithelial mesenchymal transition. Recently we found that *E*. *faecalis* can activate MMP9 secreted by macrophages in a GelE/SprE dependent manner [8]. In the present study, zymography analysis demonstrated the presence of MMP9 in its active form in the resultant conditioned media when epithelial cells were exposed to macrophage/*E*. *faecalis* (GelE/SprE) CMs (Fig 3D). To elucidate the contribution of MMP9 on the reorganization of epithelial monolayers in this model, an MMP inhibitor, (MMP9 inhibitor I, Calbichem) was used. MMP9 inhibition at a concentration of 10μM did not totally mitigate the spindle-shaped cell morphology induced by CM from J774/ *E*. *faecalis* WT (S4 Fig). It is possible that we were not able to reach an effective inhibitory dose as a higher concentration (i.e 15 μM) of the MMP9 inhibitor is toxic to epithelial cells. Alternatively, there may be other macrophage released factors (i.e growth factors, hormones, cytokines, etc) in combination with GelE/SprE, that could play a role in the observed response. It has been previously reported [6] that GelE produced by *E*. *faecalis* can compromise the epithelial barrier, however, in that study, no changes in morphotype were observed. It is likely that the interaction between *E*. *faecalis* (GelE/SprE) and macrophages is more important in this regard than the direct effect of GelE itself. One limitation of the present study is that we only exposed the apical surface of epithelial cells to the conditioned media (CM), and although this resulted in disruption of tight junctions and likely exposure of the CM to the basal surface, the extent to which this recapitulates the *in vivo* situation will require further study. A proposed model of epithelial monolayer reassembly presented on Fig 4.

We assumed that disruption of epithelial monolayer integrity begins as a process of cell extrusion whereby detached anchorage-dependent cells undergo anoikis, which is a form of programmed cell death. In the present work, staining of delaminated cells with trypan blue revealed that approximately 85% of the cells were dead, whereas the adherent cells demonstrated 98% viability. The total number delaminated cells was approximately 8.5x10^5^ and viable adherent cells ~5.2x10^5^. This indicates that approximately 38% of the epithelial cells delaminate and 62% of cells remain adherent and viable. To examine adherent cells for signs of apoptosis, epithelial cells exposed to the various macrophage/*E*. *faecalis* CMs and then were stained with FITC-annexin V. Confocal microscopy demonstrated healthy adherent cells with only a minor number of them displaying apoptosis after 18 h of incubation of the epithelial monolayer with macrophage/*E*. *faecalis* CMs (S5 Fig). Studies have demonstrated that retraction of cells can trigger coordinated elongation of neighboring cells with E-cadherin being required for both cell elongation and cell extrusion [23]. E-cadherin is often enriched in the adherent junctions of epithelial cells and can also be present in non-junctional regions of cell–cell contact [24]. These findings suggest that dynamic regulation of cadherins in response to various extracellular signals controls cell sorting, cell rearrangements and cell movements [25].

Next we performed *ex vivo* experiments to investigate the effect of J774/WT CM on mouse colon tissue explants. Explants were incubated for 6 hours with J774 CM and J774/WT CM and the apical cell surface were monitored by rhodamine wheat germ agglutinin (WGA) staining. Confocal microscopy of the luminal side of the explants demonstrated that incubation with J774/WT CM led to epithelial cell depletion and epithelial layer reorganization in comparison to J774 CM alone (S6 Fig).

### CD44^+^CD24^+^ cell populations are enriched in epithelial cells treated with macrophage/E. faecalis CMs independent of GelE/SprE

**Fig 5:**
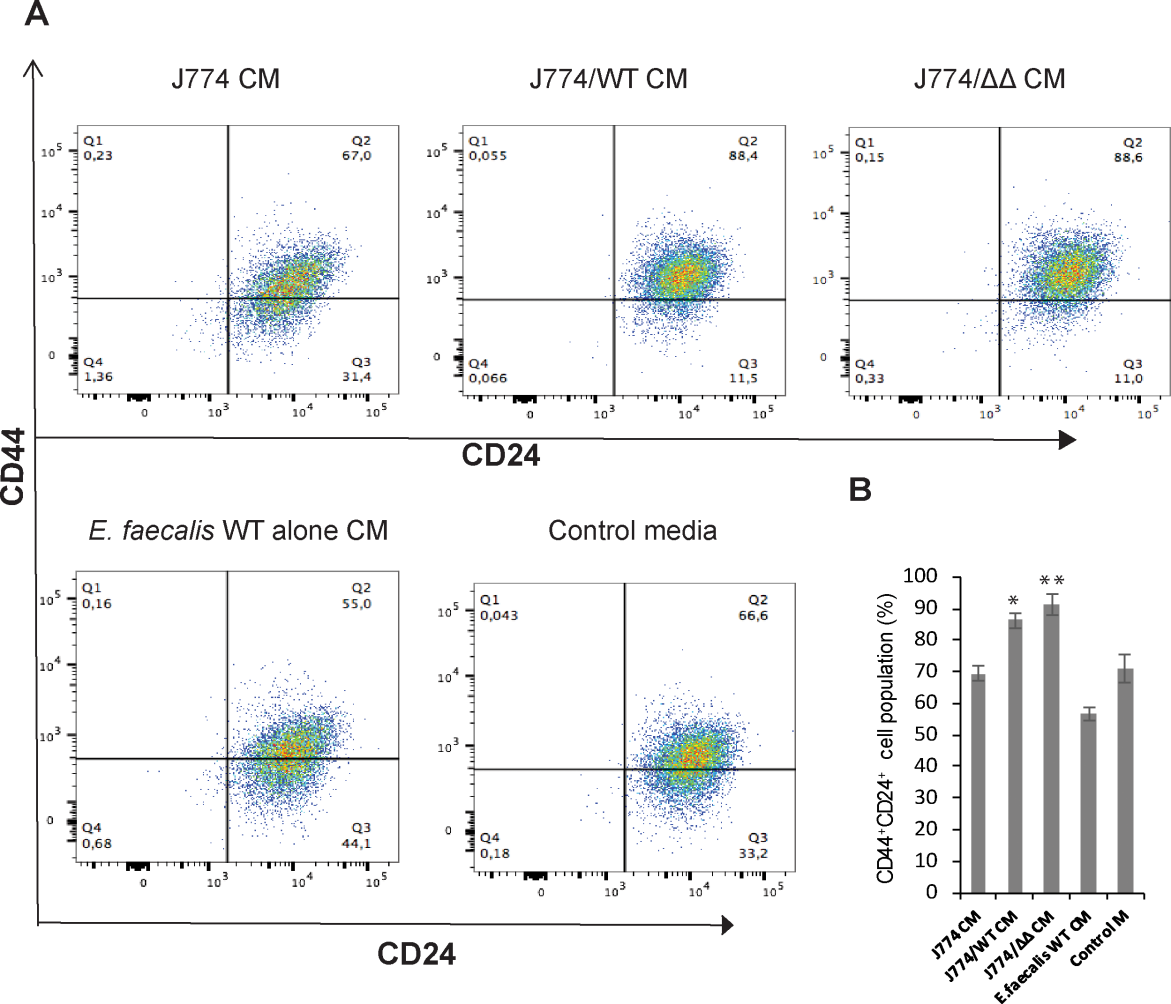
CD44^+^CD24^+^ population enriched in epithelial cells treated with J774/ *E*. *faecalis* CMs independently on GelE/SprE. **(A)** Representative results of flow cytometry analysis using CD44 and CD24 cell markers. **(B)** The percentage of CD44^+^CD24^+^ cells depending on epithelial monolayers applied CMs. Statistical analysis was performed using the one-way ANOVA with Bonferrony adjusted *p*-value for multiple comparisons where *P = 0.001, **P<0.001 as compared to J774 CM, n = 3.

In the present study, we used a primary colonic epithelial cell line, which was generated from normal mouse colon using the epithelial selective marker EpCAM-1 (CD326) according to information presented by the vender (Cell Biologics, Chicago, IL). Consequently, various types of colonic epithelial cells including stem/progenitors cells could be present in this cell line. Others have described primary colonic epithelial cell lines derived from normal human colon biopsies with progenitors cell features and the ability for self-renewal [26]. We explored the possibility that epithelial cells exposed to macrophage/*E*. *faecalis* CMs may have progenitor cell characteristics. Several studies have demonstrated that CD44^+^CD24^+^ cell populations associate with stem like behavior and the ability to self-renew [27–30]. Flow cytometry analysis for the presence of CD44 and CD24 cell surface markers, which are encoded by clusters of differentiation *CD44* and *CD24* genes, demonstrated that epithelial cells exposed to various macrophage/*E*. *faecalis* CMs differ in percentage of CD44^+^CD24^+^ cells (Fig 5A). CD44^+^CD24^+^ population are enriched in adherent cells which adopted a migrating morphology following monolayer disruption with J774/WT CM in contrast to epithelial cells held within the monolayer after incubation with J774, *E*. *faecalis* alone or control media. Interestingly, CD44^+^CD24^+^ cells are also enriched for epithelial cells treated with J774/ΔΔ CM (Fig 5B). These findings offer the possibility that the observed phenotypic changes in epithelial cells exposed to macrophage/*E*. *faecalis* CM is probably not GelE/SprE dependent, yet at the same time, disruption of monolayer integrity is strongly dependent on GelE/SprE. Our findings suggest that, following disruption of the monolayers, progenitor cells undergo morphological transformation and acquire a migrating phenotype. However, we cannot exclude the possibility that non-stem epithelial cells acquire a migrating phenotype with stem like characteristics.

### Single epithelial cells C57/J774/WT form distinctly shaped self-organizing cysts in matrigel

**Fig 6:**
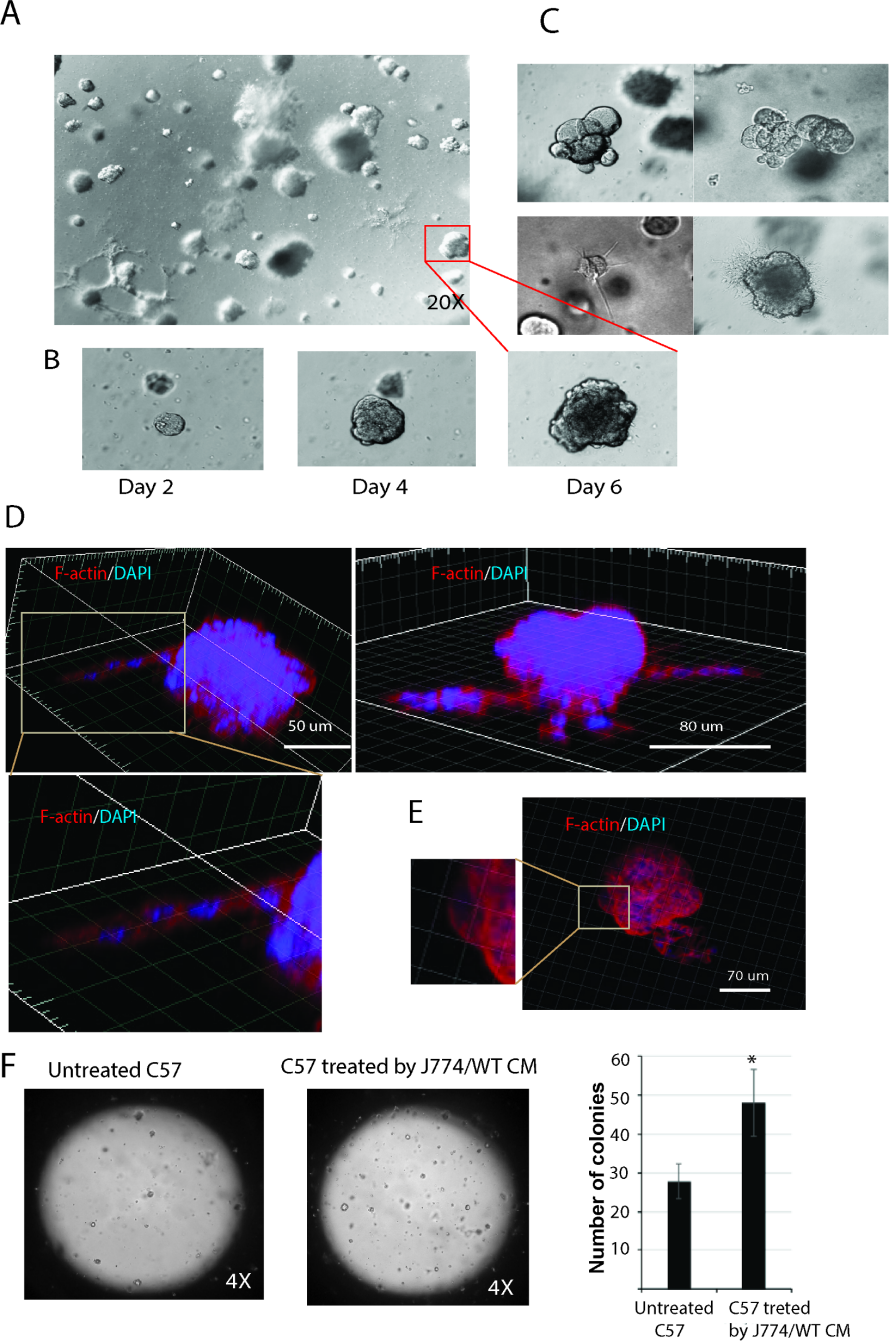
C57/J774/WT cells form distinct type of colonies in matrigel. **(A)** Phase contrast images of colonies originated from a single cell suspension of adherent C57/J774/WT cells after 6 days growth in 3D matrigel. **(B)** Dynamic growth one colony in 3D matrigel by days; **(C)** Phase contrast images demonstrate different type of colonies as round colonies (upper panel) and colonies with branching (lower panel). **(D, E)** Reconstructed 3D images of single colonies stained for F-actin (phalloidin, red) and nuclei (DAPI, blue). Images demonstrate **(D)** cellular extensions and **(E)** actin-rich cellular protrusions. **(F)** Epithelial cell suspension in matrigel (50 μl containing ~ 500 cells) was plated into 1 well of an 8-well camber-slide and grown in medium supplemented with 10% FBS. After 1 week of incubation, the colonies were identified. C57cells treated with J774/WT CM give rise to higher number of colonies compared to untreated cells. Statistical analysis was performed using the one-way ANOVA where *P = 0.023 as compared to untreated C57 cells, n = 3.

To test the functionality of transformed cells to self-renew, a single-cell suspension of adherent cells following detachment with trypsin and filtration were embedded onto 3-D matrigel. Cells were grown in serum-supplemented media in the absence of feeder layers to assess for their multipotent capacity. After 6 days, cells were observed to give rise to multicellular structures in the matrigel (Fig 6A). The typical growth of one self-organizing cyst following 6 days is shown in Fig 6B. These results indicate that formation of various types of multicellular structures (Fig 6C) such as compact (upper panel) or with branching chains of cells extensions invading into the matrigel (lower panel) can be observed under these experimental conditions. To assess the spatial organization of multicellular structures in the 3D culture, F-actin staining was performed. Results of the 3D projected images demonstrated the presence of branching chains of cells (Fig 6D) and the presence of actin-rich cellular protrusions (Fig 6E). Quantification of multicellular structures growing in matrigel demonstrated that C57cells treated with J774/WT CM give rise to a higher number of colonies compared to untreated cells (Fig 6F).

In summary, results from the series of experiments in this study demonstrate that the interaction of the commensal bacteria *E*. *faecalis* and macrophages results in a conditioned media (CM) that causes reassembly of epithelial monolayers and promotes epithelial cells to express a migrating phenotype. Such reorganization of epithelial monolayers by the macrophage/*E*. *faecalis* CM suggests that cell migration following monolayer disruption may give rise to self-organizing cyst formation. These findings may have important implications for inflammatory, injured and malignant conditions of the intestine.

## Supporting information

S1 Fig *E*. *faecalis* WT strain growth in co-culture with J774 macrophages at MOI = 5 during 1-5h. After 5h of incubation with macrophages, CFUs of *E*. *faecalis* WT were increased 3 fold. CFUs were determined by plating samples by serial dilution. Data are combined from 2 independent experiments; mean values are graphed and error bars represent standard error of the mean (SEM).
(PDF)

S2 Fig LDH activity is negligible following infection of J774 macrophages with *E*. *faecalis* WT at MOI = 5 for 5h. LDH positive control (Promega) for cell death. Data are combined from 3 independent experiments; mean values are graphed and error bars represent standard error of the mean (SEM).
(PDF)

S3 Fig Western blot data from 3 independent experiments to identify E-cadherin in cells after 18 h treatment of epithelial cell monolayers with macrophage/E. faecalis CM. (PDF)

S4 Fig MMP9 inhibitor I (Calbiochem) does not completely abrogate morphological changes initiated by J774/WT CM in C57 epithelial cells. **(A)** Phase contrast microscopy of epithelial cells treated by J774/WT in presence (10μM) or absence of MMP9 inhibitor I. **(B)** The percentage of spindle-shaped cells in C57/B6 cells treated by J774/WT with or without MMP9 Inhibitor I. The difference between J774/WT versus J774/WT + MMP9 inhibitor I was not statistically different. Statistical analysis was performed using the one-way ANOVA where *P = 0.078 as compared to J774/WT CM, n = 3.
(PDF)

S5 Fig Adherent epithelial cells do not undergo apoptosis in response to conditioned media from J774 macrophages exposed to the various *E*. *faecalis* strains. Confocal images of epithelial cells stained for apoptosis with annexin V (green) after 18 h of incubation with J774/*E*. *faecalis* CMs. Nuclei stained with Hoechst 33342 (blue).
(PDF)

S6 Fig Incubation of colon tissue explants with J774/WT CM leads to depletion of epithelial cells and reorganization of the luminal side of tissues. Colon tissue explants stained with the epithelial marker rhodamine labelled WGA (red) and nuclei stained with DAPI (blue) after incubation for 6 h with **(A)** control J774 CM and **(B)** J774/WT CM.
(PDF)

## References

[1] Artis D. Epithelial-cell recognition of commensal bacteria and maintenance of immune homeostasis in the gut. Nature reviews Immunology. 2008;8(6):411–20. Epub 2008/05/13. doi: 10.1038/nri2316 18469830 10.1038/nri2316

[2] Terzic J, Grivennikov S, Karin E, Karin M. Inflammation and colon cancer. Gastroenterology. 2010;138(6):2101–14 e5. Epub 2010/04/28. doi: 10.1053/j.gastro.2010.01.058 20420949 10.1053/j.gastro.2010.01.058

[3] Rieder F. The gut microbiome in intestinal fibrosis: environmental protector or provocateur? Science translational medicine. 2013;5(190):190ps10 Epub 2013/06/21. doi: 10.1126/scitranslmed.3004731 23785034 10.1126/scitranslmed.3004731 PMC3823049

[4] Umar S. Enteric pathogens and cellular transformation: bridging the gaps. Oncotarget. 2014;5(16):6573–5. Epub 2014/09/13. doi: 10.18632/oncotarget.2384 25216513 10.18632/oncotarget.2384 PMC4196145

[5] Steck N, Mueller K, Schemann M, Haller D. Bacterial proteases in IBD and IBS. Gut. 2012;61(11):1610–8. Epub 2011/09/09. doi: 10.1136/gutjnl-2011-300775 21900548 10.1136/gutjnl-2011-300775

[6] Steck N, Hoffmann M, Sava IG, Kim SC, Hahne H, Tonkonogy SL, et al Enterococcus faecalis metalloprotease compromises epithelial barrier and contributes to intestinal inflammation. Gastroenterology. 2011;141(3):959–71. Epub 2011/06/28. doi: 10.1053/j.gastro.2011.05.035 21699778 10.1053/j.gastro.2011.05.035

[7] Ocvirk S, Sava IG, Lengfelder I, Lagkouvardos I, Steck N, Roh JH, et al Surface-Associated Lipoproteins Link Enterococcus faecalis Virulence to Colitogenic Activity in IL-10-Deficient Mice Independent of Their Expression Levels. PLoS pathogens. 2015;11(6):e1004911 Epub 2015/06/13. doi: 10.1371/journal.ppat.1004911 26067254 10.1371/journal.ppat.1004911 PMC4466351

[8] Shogan BD, Belogortseva N, Luong PM, Zaborin A, Lax S, Bethel C, et al Collagen degradation and MMP9 activation by Enterococcus faecalis contribute to intestinal anastomotic leak. Science translational medicine. 2015;7(286):286ra68 Epub 2015/05/08. doi: 10.1126/scitranslmed.3010658 25947163 10.1126/scitranslmed.3010658 PMC5027898

[9] Thomas VC, Thurlow LR, Boyle D, Hancock LE. Regulation of autolysis-dependent extracellular DNA release by Enterococcus faecalis extracellular proteases influences biofilm development. Journal of bacteriology. 2008;190(16):5690–8. Epub 2008/06/17. doi: 10.1128/JB.00314-08 18556793 10.1128/JB.00314-08 PMC2519388

[10] Whitehead RH, Robinson PS. Establishment of conditionally immortalized epithelial cell lines from the intestinal tissue of adult normal and transgenic mice. American journal of physiology Gastrointestinal and liver physiology. 2009;296(3):G455–60. Epub 2008/12/26. doi: 10.1152/ajpgi.90381.2008 19109407 10.1152/ajpgi.90381.2008 PMC2660172

[11] O'Rourke KP, Dow LE, Lowe SW. Immunofluorescent Staining of Mouse Intestinal Stem Cells. Bio-protocol. 2016;6(4). Epub 2016/08/30. 10.21769/bioprotoc.1732 PMC4996654 27570798

[12] Heussen C, Dowdle EB. Electrophoretic analysis of plasminogen activators in polyacrylamide gels containing sodium dodecyl sulfate and copolymerized substrates. Analytical biochemistry. 1980;102(1):196–202. Epub 1980/02/01. 7188842 10.1016/0003-2697(80)90338-3

[13] Mendez MG, Kojima S, Goldman RD. Vimentin induces changes in cell shape, motility, and adhesion during the epithelial to mesenchymal transition. FASEB journal: official publication of the Federation of American Societies for Experimental Biology. 2010;24(6):1838–51. Epub 2010/01/26. 20097873 10.1096/fj.09-151639 PMC2874471

[14] Tsai JH, Yang J. Epithelial-mesenchymal plasticity in carcinoma metastasis. Genes & development. 2013;27(20):2192–206. Epub 2013/10/22. 24142872 10.1101/gad.225334.113 PMC3814640

[15] Arjonen A, Kaukonen R, Ivaska J. Filopodia and adhesion in cancer cell motility. Cell adhesion & migration. 2011;5(5):421–30. Epub 2011/10/07. 21975551 10.4161/cam.5.5.17723 PMC3218609

[16] Yamaguchi H, Condeelis J. Regulation of the actin cytoskeleton in cancer cell migration and invasion. Biochimica et biophysica acta. 2007;1773(5):642–52. Epub 2006/08/24. doi: 10.1016/j.bbamcr.2006.07.001 16926057 10.1016/j.bbamcr.2006.07.001 PMC4266238

[17] Johnson HE, King SJ, Asokan SB, Rotty JD, Bear JE, Haugh JM. F-actin bundles direct the initiation and orientation of lamellipodia through adhesion-based signaling. The Journal of cell biology. 2015;208(4):443–55. Epub 2015/02/11. doi: 10.1083/jcb.201406102 25666809 10.1083/jcb.201406102 PMC4332254

[18] Turner JR. Intestinal mucosal barrier function in health and disease. Nature reviews Immunology. 2009;9(11):799–809. Epub 2009/10/27. doi: 10.1038/nri2653 19855405 10.1038/nri2653

[19] Nava P, Kamekura R, Nusrat A. Cleavage of transmembrane junction proteins and their role in regulating epithelial homeostasis. Tissue barriers. 2013;1(2):e24783 Epub 2014/03/26. doi: 10.4161/tisb.24783 24665393 10.4161/tisb.24783 PMC3879235

[20] Suzuki T. Regulation of intestinal epithelial permeability by tight junctions. Cellular and molecular life sciences: CMLS. 2013;70(4):631–59. Epub 2012/07/12. doi: 10.1007/s00018-012-1070-x 22782113 10.1007/s00018-012-1070-x PMC11113843

[21] Symowicz J, Adley BP, Gleason KJ, Johnson JJ, Ghosh S, Fishman DA, et al Engagement of collagen-binding integrins promotes matrix metalloproteinase-9-dependent E-cadherin ectodomain shedding in ovarian carcinoma cells. Cancer research. 2007;67(5):2030–9. Epub 2007/03/03. doi: 10.1158/0008-5472.CAN-06-2808 17332331 10.1158/0008-5472.CAN-06-2808

[22] Zheng G, Lyons JG, Tan TK, Wang Y, Hsu TT, Min D, et al Disruption of E-cadherin by matrix metalloproteinase directly mediates epithelial-mesenchymal transition downstream of transforming growth factor-beta1 in renal tubular epithelial cells. The American journal of pathology. 2009;175(2):580–91. Epub 2009/07/11. doi: 10.2353/ajpath.2009.080983 19590041 10.2353/ajpath.2009.080983 PMC2716958

[23] Grieve AG, Rabouille C. Extracellular cleavage of E-cadherin promotes epithelial cell extrusion. Journal of cell science. 2014;127(Pt 15):3331–46. Epub 2014/06/05. doi: 10.1242/jcs.147926 24895403 10.1242/jcs.147926

[24] Levi G, Gumbiner B, Thiery JP. The distribution of E-cadherin during Xenopus laevis development. Development. 1991;111(1):159–69. Epub 1991/01/01. 2015791 10.1242/dev.111.1.159

[25] Gumbiner BM. Regulation of cadherin-mediated adhesion in morphogenesis. Nature reviews Molecular cell biology. 2005;6(8):622–34. Epub 2005/07/19. doi: 10.1038/nrm1699 16025097 10.1038/nrm1699

[26] Roig AI, Eskiocak U, Hight SK, Kim SB, Delgado O, Souza RF, et al Immortalized epithelial cells derived from human colon biopsies express stem cell markers and differentiate in vitro. Gastroenterology. 2010;138(3):1012–21 e1-5. Epub 2009/12/08. doi: 10.1053/j.gastro.2009.11.052 19962984 10.1053/j.gastro.2009.11.052

[27] Wang F, Scoville D, He XC, Mahe MM, Box A, Perry JM, et al Isolation and characterization of intestinal stem cells based on surface marker combinations and colony-formation assay. Gastroenterology. 2013;145(2):383–95 e1-21. Epub 2013/05/07. doi: 10.1053/j.gastro.2013.04.050 23644405 10.1053/j.gastro.2013.04.050 PMC3781924

[28] Gracz AD, Fuller MK, Wang F, Li L, Stelzner M, Dunn JC, et al Brief report: CD24 and CD44 mark human intestinal epithelial cell populations with characteristics of active and facultative stem cells. Stem Cells. 2013;31(9):2024–30. Epub 2013/04/05. doi: 10.1002/stem.1391 23553902 10.1002/stem.1391 PMC3783577

[29] Yeung TM, Gandhi SC, Wilding JL, Muschel R, Bodmer WF. Cancer stem cells from colorectal cancer-derived cell lines. Proceedings of the National Academy of Sciences of the United States of America. 2010;107(8):3722–7. Epub 2010/02/06. doi: 10.1073/pnas.0915135107 20133591 10.1073/pnas.0915135107 PMC2840416

[30] Okabe H, Ishimoto T, Mima K, Nakagawa S, Hayashi H, Kuroki H, et al CD44s signals the acquisition of the mesenchymal phenotype required for anchorage-independent cell survival in hepatocellular carcinoma. British journal of cancer. 2014;110(4):958–66. Epub 2013/12/05. doi: 10.1038/bjc.2013.759 24300972 10.1038/bjc.2013.759 PMC3929866

