# Supplementary figures and images for: Media from macrophages co-incubated with Enterococcus faecalis induces epithelial cell monolayer reassembly and altered cell morphology
Source: PLoS One. 2017 Aug 9;12(8):e0182825. doi: 10.1371/journal.pone.0182825 (PMC5549984; doi:10.1371/journal.pone.0182825)

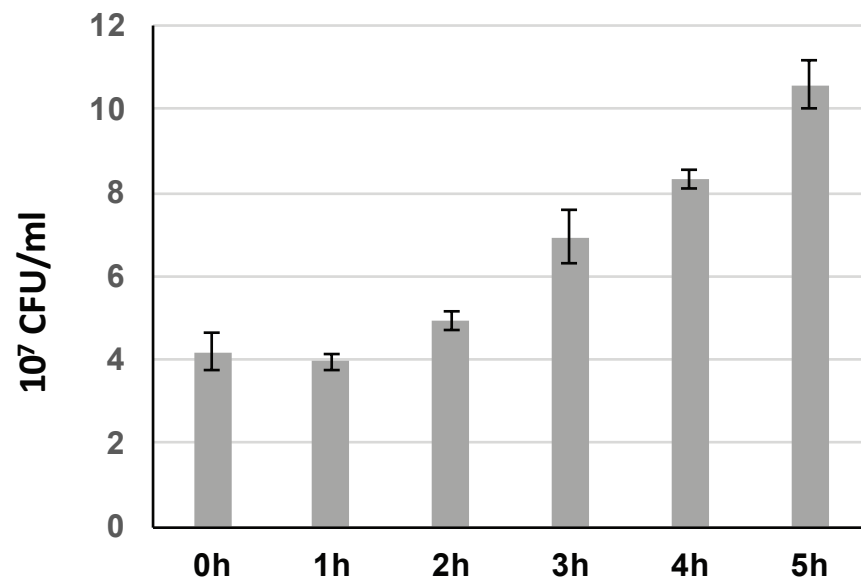

**S1 Fig.**

Supplement: S1 Fig — After 5h of incubation with macrophages, CFUs of E. faecalis WT were increased 3 fold. CFUs were determined by plating samples by serial dilution. Data are combined from 2 independent experiments; mean values are graphed and error bars represent standard error of the mean (SEM). (PDF) [file pone.0182825.s001.pdf]

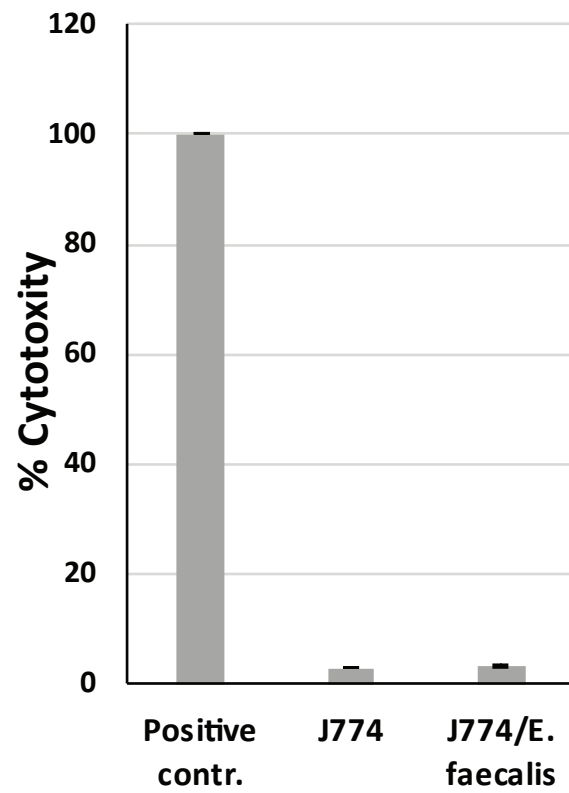

**S2 Fig.**

Supplement: S2 Fig — LDH positive control (Promega) for cell death. Data are combined from 3 independent experiments; mean values are graphed and error bars represent standard error of the mean (SEM). (PDF) [file pone.0182825.s002.pdf]

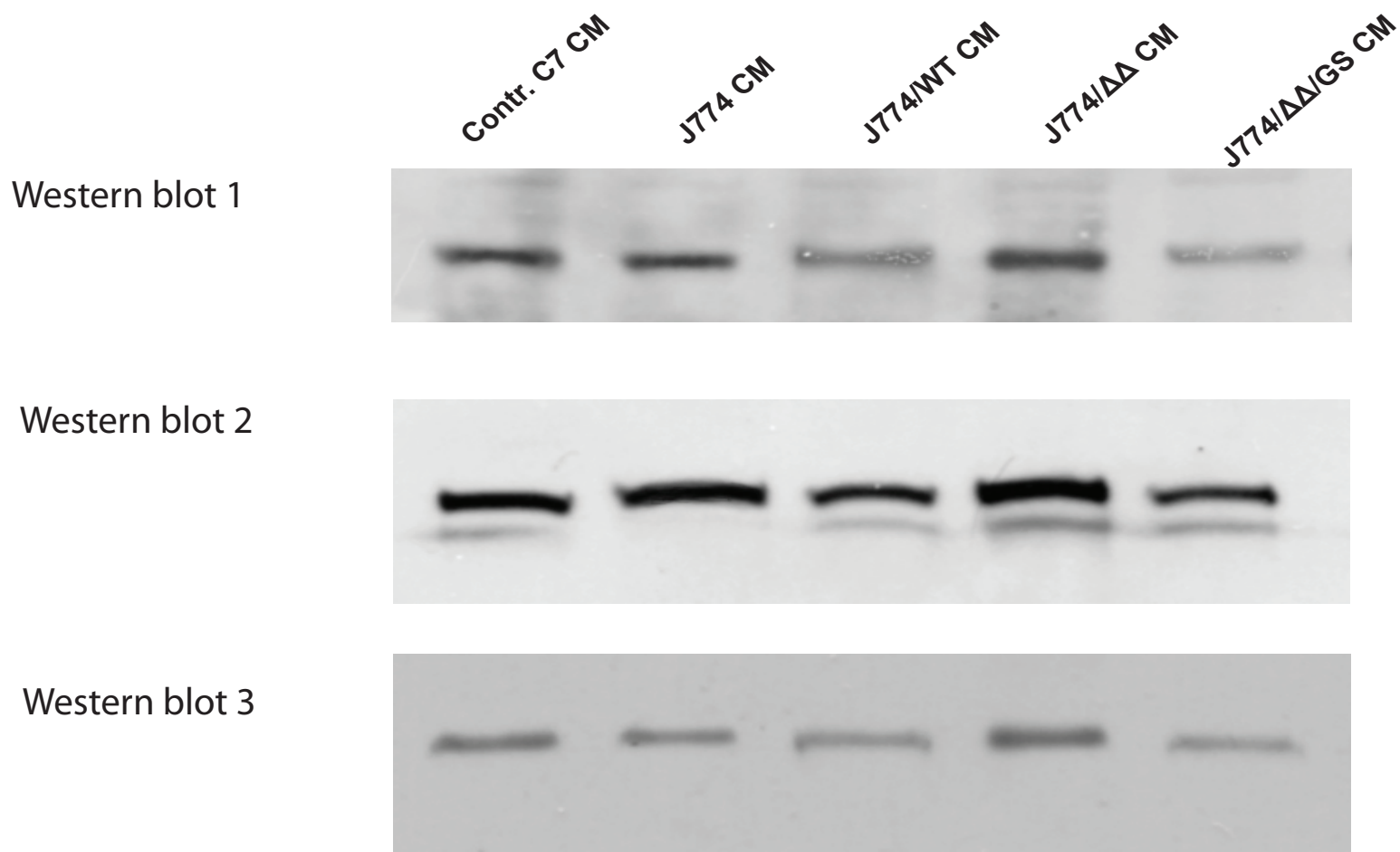

**S3 Fig.**

Supplement: S3 Fig — (PDF) [file pone.0182825.s003.pdf]

**A**

**J774 CM**

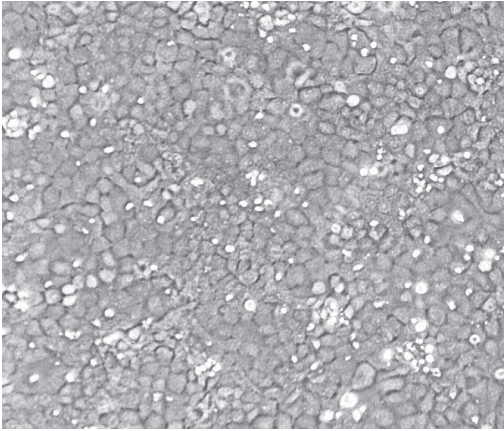

**J774/WT CM**

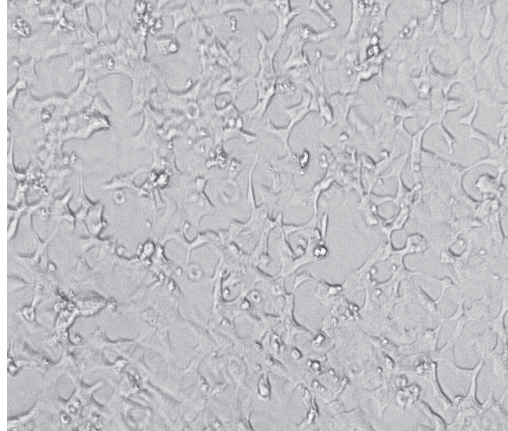

**J774/WT + MMP9 Inhibitor I**

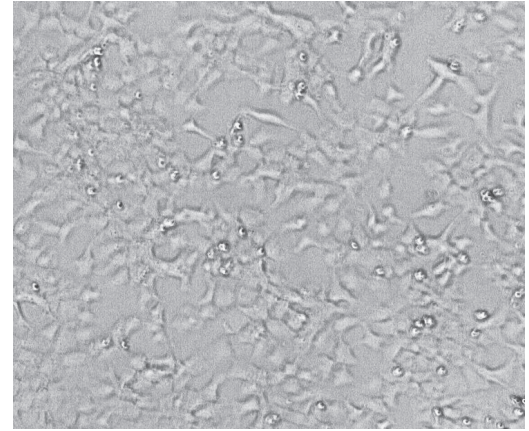

**B**

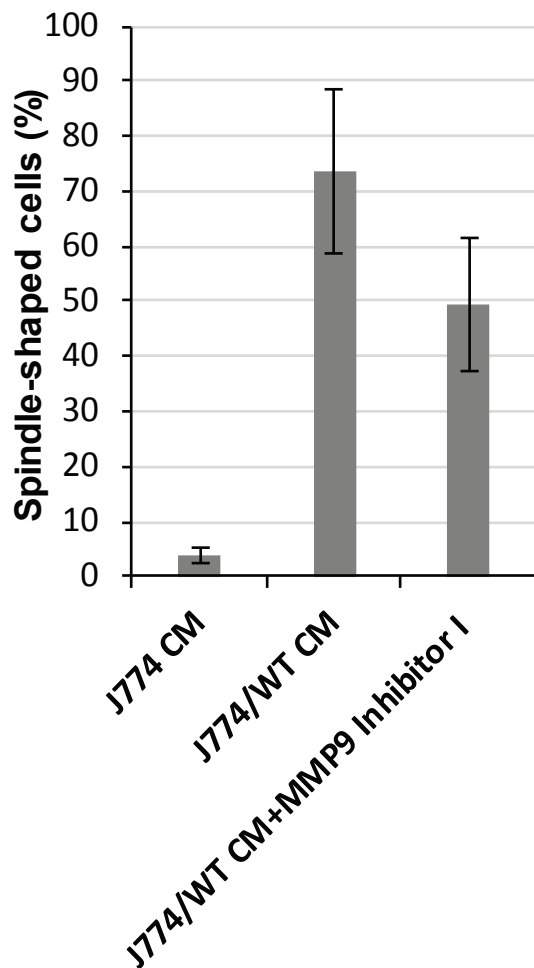

**S4 Fig.**

Supplement: S4 Fig — (A) Phase contrast microscopy of epithelial cells treated by J774/WT in presence (10μM) or absence of MMP9 inhibitor I. (B) The percentage of spindle-shaped cells in C57/B6 cells treated by J774/WT with or without MMP9 Inhibitor I. The difference between J774/WT versus J774/WT + MMP9 inhibitor I was not statistically different. Statistical analysis was performed using the one-way ANOVA where *P = 0.078 as compared to J774/WT CM, n = 3. (PDF) [file pone.0182825.s004.pdf]

**J774 CM**

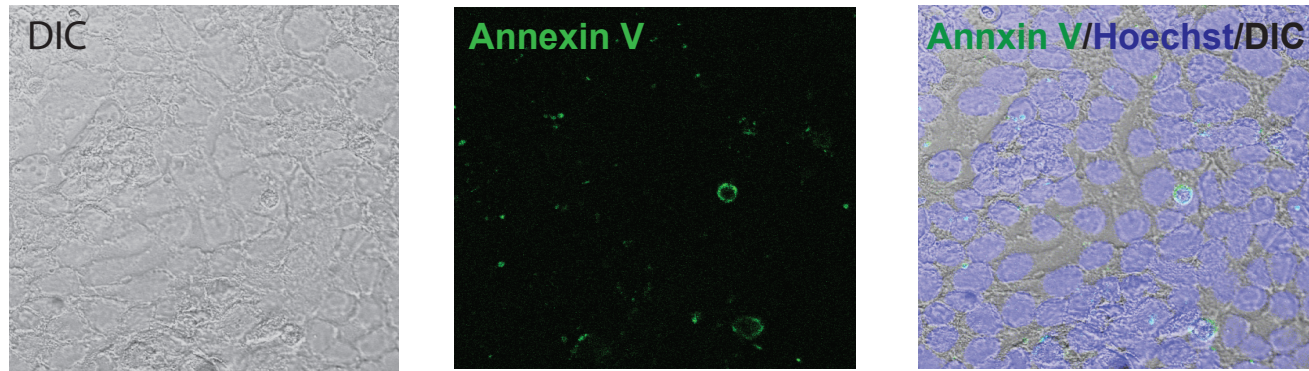

**J774/WT CM**

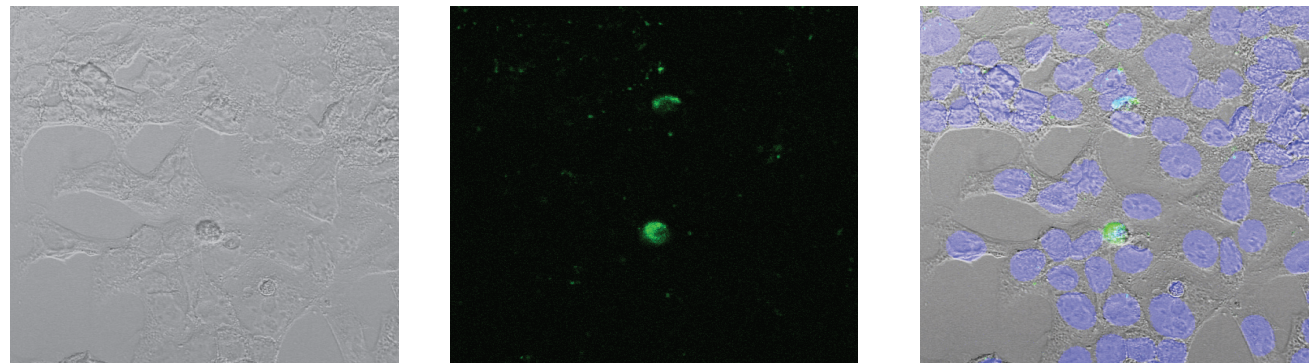

**J774/ $\Delta\Delta$  CM**

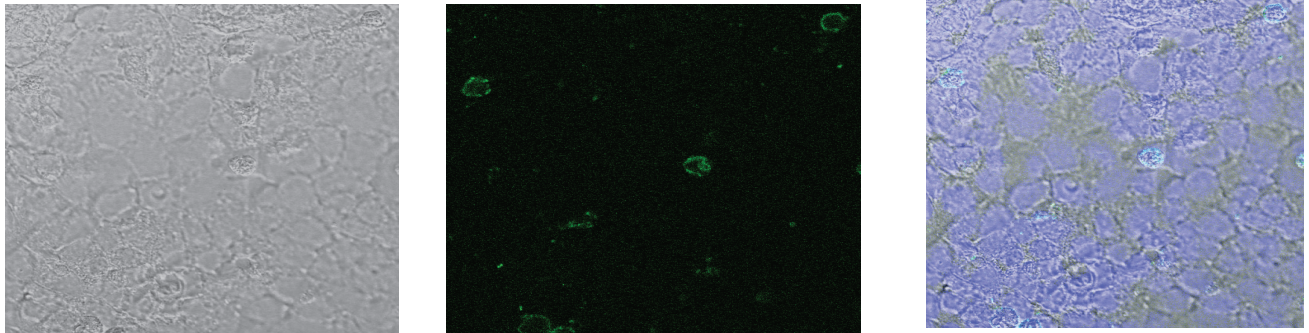

**J774/ $\Delta\Delta$ /GS CM**

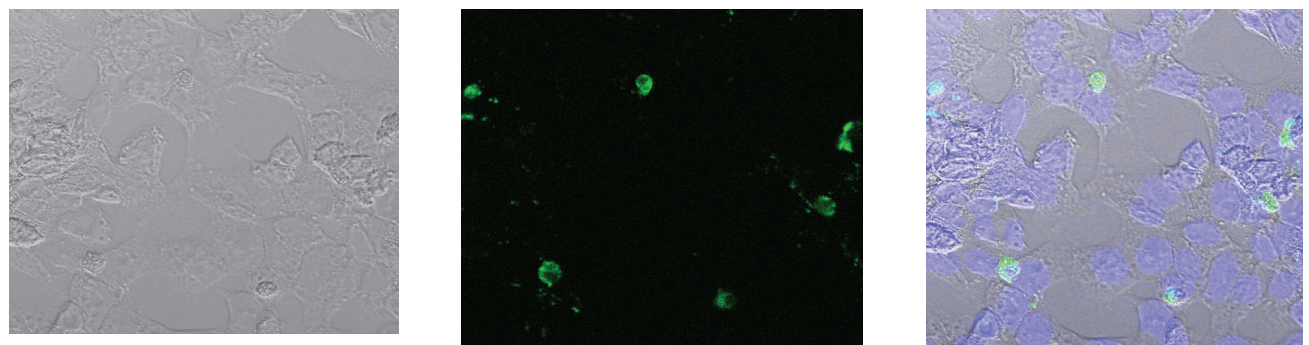

**S5 Fig.**

Supplement: S5 Fig — Confocal images of epithelial cells stained for apoptosis with annexin V (green) after 18 h of incubation with J774/E. faecalis CMs. Nuclei stained with Hoechst 33342 (blue). (PDF) [file pone.0182825.s005.pdf]

**A**

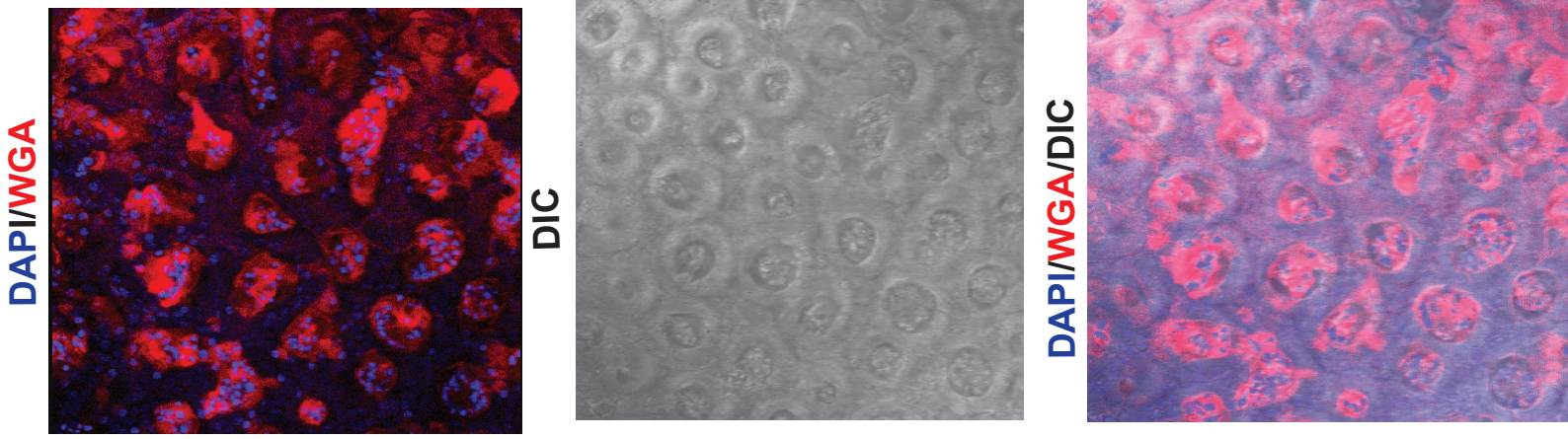

**B**

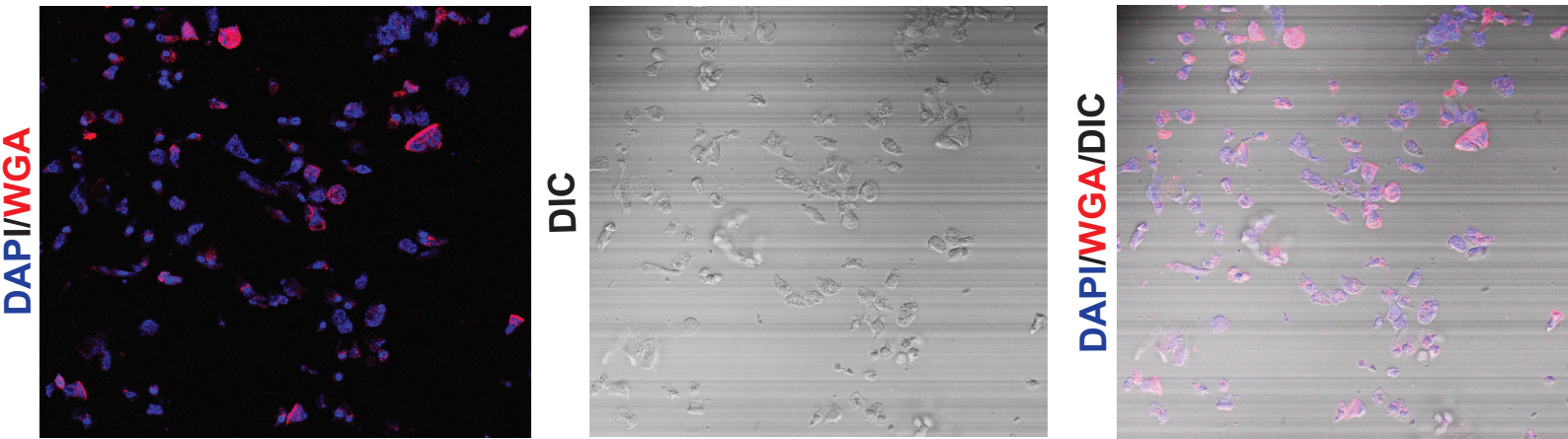

**S6 Fig.**

Supplement: S6 Fig — Colon tissue explants stained with the epithelial marker rhodamine labelled WGA (red) and nuclei stained with DAPI (blue) after incubation for 6 h with (A) control J774 CM and (B) J774/WT CM. (PDF) [file pone.0182825.s006.pdf]
